# The development of a return to work intervention programme for stroke survivor (SReTWIP): a Delphi survey

**DOI:** 10.1186/s12883-020-01668-6

**Published:** 2020-03-13

**Authors:** Olumide Ayoola Olaoye, Shaheed Moghammad Soeker, Anthea Rhoda

**Affiliations:** 1 Departments of Occupational Therapy, Faculty of Community and Health Sciences, University of the Western Cape, Cape Town, South Africa; 2 Departments of Medical Rehabilitation, College of Health Sciences, Obafemi Awolowo University, Ile-Ife, Nigeria; 3 Department of Physiotherapy, Faculty of Community and Health Sciences, University of the Western Cape, Cape Town, South Africa

**Keywords:** Stroke, Vocational rehabilitation, Return to work, Intervention, Programme, Cerebrovascular accident

## Abstract

**Background:**

Even though clearly defined pathways for vocational re-entry are well recognized for conditions such as mental health, musculoskeletal dysfunction (MSD) and traumatic brain injury (TBI), none has been identified for stroke. There has been a lack of consensus regarding such clear pathways to vocational re-entry and the essential contents of return to work (RTW) interventions for stroke survivors. As part of a larger study aimed to design a RTW programme for stroke survivors, this study describes the concluding process through which Stroke Return to Work Intervention Programme (SReTWIP) was developed.

**Methods:**

Experts in the field of neurorehabilitation and vocational rehabilitation (VR) from 6 countries participated in this 3-round Delphi survey via e-mail. Concept mapping was used to triangulate findings from the Delphi with previous phases of the larger study. Content thematic analysis was conducted on qualitative data while descriptive statistic was used to analyze quantitative data.

**Results:**

Fifteen experts with a mean age and mean duration of practice of 44.73 ± 9.48 years and 18.26 ± 8.71 years respectively participated in this study. The developed RTW programme (SReTWIP) is a 12 week programme that consisted of four interconnected phases of intervention viz.: an assessment phase, clinic-based work and non-work specific intervention phase, a work test placement phase and a client full participation in worker role phase. The experts agreed on a set of implementation strategies that included the use of interdisciplinary team, the tailoring of intervention to meet stroke survivor’s need, as well as the use of case management approach.

**Conclusion:**

The SReTWIP is the first step in developing a VR pathway that can ultimately enhance the RTW rates and quick resumption of the worker role of stroke survivors. The stroke survivor can move along the different phases of the SReTWIP after achieving competency in a preceding phase. Future work will include a feasibility study with other key stakeholders involved in RTW such as employers, informal caregivers and stroke survivors before its implementation.

## Background of the study

Over the past decades, there has been growing concern on the increasing incidence and prevalence of stroke and its attendant high morbidity in developing countries [1, 2]. Arising from acute focal or global injury to the central nervous system by a vascular cause, stroke could result in considerable neurological deficits on its survivor [2]. After a stroke, significant number of survivors experience prolonged work absence that render them unproductive within the community. Meanwhile, it is documented that RTW after prolong work absence due to ill health requires a well-defined VR pathway [3, 4]. This had resulted in emergent interest in the application of evidence-based RTW interventions for work disability management for various health conditions. For instance, supported employment programme and model of occupational self-efficacy are evidence-based VR programmes that have over the years been used to facilitate work integration for individuals with serious mental illness and TBI respectively [5, 6]. Even though clearly defined pathways for vocational re-entry are well recognized for chronic conditions such as mental health, MSD and TBI [5–7], none has been identified for stroke as there has been a lack of consensus regarding such clear pathways to vocational re-entry. Similarly, no validated evidence has been established for the essential contents of RTW interventions needed to effectively facilitate work reintegration for the individuals that have experienced stroke. More specifically, in Nigeria which serve as the study context, there is a gap in the strategies to facilitate work re-entry for stroke survivors. Although, mental illness, MSD and TBI are chronic conditions, stroke is an acute disease with a potential chronic sequelae that influences work functioning. As part of a larger study aimed to design a RTW programme for stroke survivors, a preliminary study (Study I) on need assessment and identification of performance objective was conducted [8]. The study surveyed 210 stroke survivors in south-west, Nigeria using the Work Rehabilitation Questionnaire, the International Classification of Functioning, Disability and Health (ICF) Brief Core Sets for VR and the Work Impact Questionnaire to establish baseline data on the impairments, activity limitation and participation restrictions experienced by stroke survivors, as well as their RTW rates [8]. The study found that more than a third of stroke survivors did not RTW as a result of the sequelae of stroke while only half of the survivors that RTW did so at a reduced capacity (part-time and light duty). Similarly, the above study discovered patterns across marital status, disability level, work category and workplace support in how stroke survivors RTW. While identifying the performance objectives that were needed to facilitate RTW of stroke survivors in the same study, it was established that the side of stroke affectation, type of rehabilitation programme, stroke symptoms, environmental factor as well as problem experienced by survivors in activity and participation could significantly predict RTW capacity of stroke survivors. It was also found that work resumption after stroke was influenced by the recovery of functional abilities of the survivor; access to rehabilitation services; workplace directed interventions; as well as self-determination of the stroke survivor to RTW.

Similarly, as part of the larger study, a review of literature (Study II) was conducted to identify effective RTW interventions for stroke survivors [9]. The above study reviewed 32 articles and one clinical guideline from nine databases and grey literature that transverse 11-year period using the Arskey and O’Malley scoping review methodology [10]. The reviewed studies vary in terms of rationale, methodology design, description of intervention activities, and period of deployment and delivery mode of interventions. Three core components of RTW interventions that included intervention components that interfaced with the stroke survivor, intervention components that interfaced with the workplace and, implementation strategies were identified [9].

In order to arrive at a consensus regarding the RTW intervention components that are essential and to define a clear vocational pathway for implementing such RTW components when replicating effective interventions, this final stage of the study was conducted. This study describes the process through which the SReTWIP was developed.

## Methods

For the development of the SReTWIP, the authors used a multi-phase mixed method research approach that was guided by Intervention Mapping framework [11]. This comprised three iterative studies that informed one another with the findings culminating into the SReTWIP. The concluding study (Study III-Delphi survey) which attempts to identify the essential components of RTW intervention and to define a clear vocational pathway for implementing such RTW components when replicating effective interventions is described in this manuscript. Delphi survey [12, 13] is a recognized consensus formation method which involves the achievement of consensus among a group of experts via series of survey. While the first survey is usually open ended, the subsequent surveys are shaped by the results of the prior ones. A modified e-Delphi survey was utilized in this study The modification to the e-Delphi entailed merging of the opinion of experts from the first round of the Delphi with evidences from the previous studies [8, 9] for further controlled feedbacks in the subsequent rounds of the Delphi process. The participants were experts in the field of neuro-rehabilitation and VR involved in the RTW process of stroke survivors. They were purposively selected from healthcare circle and academia.

### Delphi rounds

A peculiar feature of the Delphi survey is the multiple iterations that entail a series of feedback processes which allow experts to review their opinions on a topic [14]. In the present study, the e-Delphi survey consisted of three rounds conducted over a 6 month period. Consensus was reached at the end of the third round of the e-Delphi.

### Selection of experts

In order to capture diverse knowledge and opinion pertaining to RTW interventions for stroke survivors, the Delphi selection process described by Okoli and Pawloski [15] was used to purposively select 29 experts comprising occupational therapists (OT), physiotherapists (PT), and clinical psychologists who were familiar with the Nigerian health context. Experts without prior knowledge of the study context were provided with adequate information to guide with decision making during the survey.

### Procedure

The Research Ethics and Higher Degrees Committee of the University of the Western Cape, South Africa as well as the Health Research and Ethics Committee of the Institute of Public Health, Obafemi Awolowo University, Nigeria, gave approval for this study. Online invitation, consent and information sheet was sent out to 29 selected experts. Eighteen of the experts initially provided written consent to participate with three later recusing themselves for lack of adequate knowledge on the topic of interest. No reason was given by the other experts who declined to participate in the study. Consenting experts responded to an online questionnaire that sought information on their demographic profile. The first round of the survey spanned 4 weeks during which 15 experts provided answers to four open ended questions regarding interventions that were needed to facilitate RTW of stroke survivors. At the end of the first round, opinions were collated and analyzed. Results obtained were triangulated with findings from the previous phases (Study I and Study II) of the study into an initial concept map that was later transformed into a draft SReTWIP. The second round entailed presenting the draft SReTWIP to the experts to rate its feasibility on a three point nominal scale of disagree; indifferent; and agree. They were also requested to provide further input on the content and structure, components, approaches, implementation strategies and duration of implementation of the programme. In the third round, questions without consensus were reviewed base on input from experts and pulled into a questionnaire which was sent back to the experts. These included items that had between five to eight experts that agreed to their inclusion and less than 5 experts that agreed to their exclusion.

They were requested to indicate the appropriateness of the revisions made to the intervention contents using binary options. Base on the suggestions provided by the experts, the duration of the phases of intervention was reviewed and divided into ‘how long’ (timing) and “how often” (the numbers of sessions required). To be eligible for inclusion in the SReTWIP, consensus was set at 69% and above for the second and third rounds. The experts were thereafter notified of the completion of the final round of the Delphi and provided with a copy of the developed SReTWIP.

### Data analysis

The data obtained from the first round of the Delphi survey was analyzed thematically using the procedure described by Corbin and Strauss [16]. The quantitative responses from the experts in the second and third round were analyzed descriptively using means of central tendency and percentages.

## Results

**Fig. 1:**
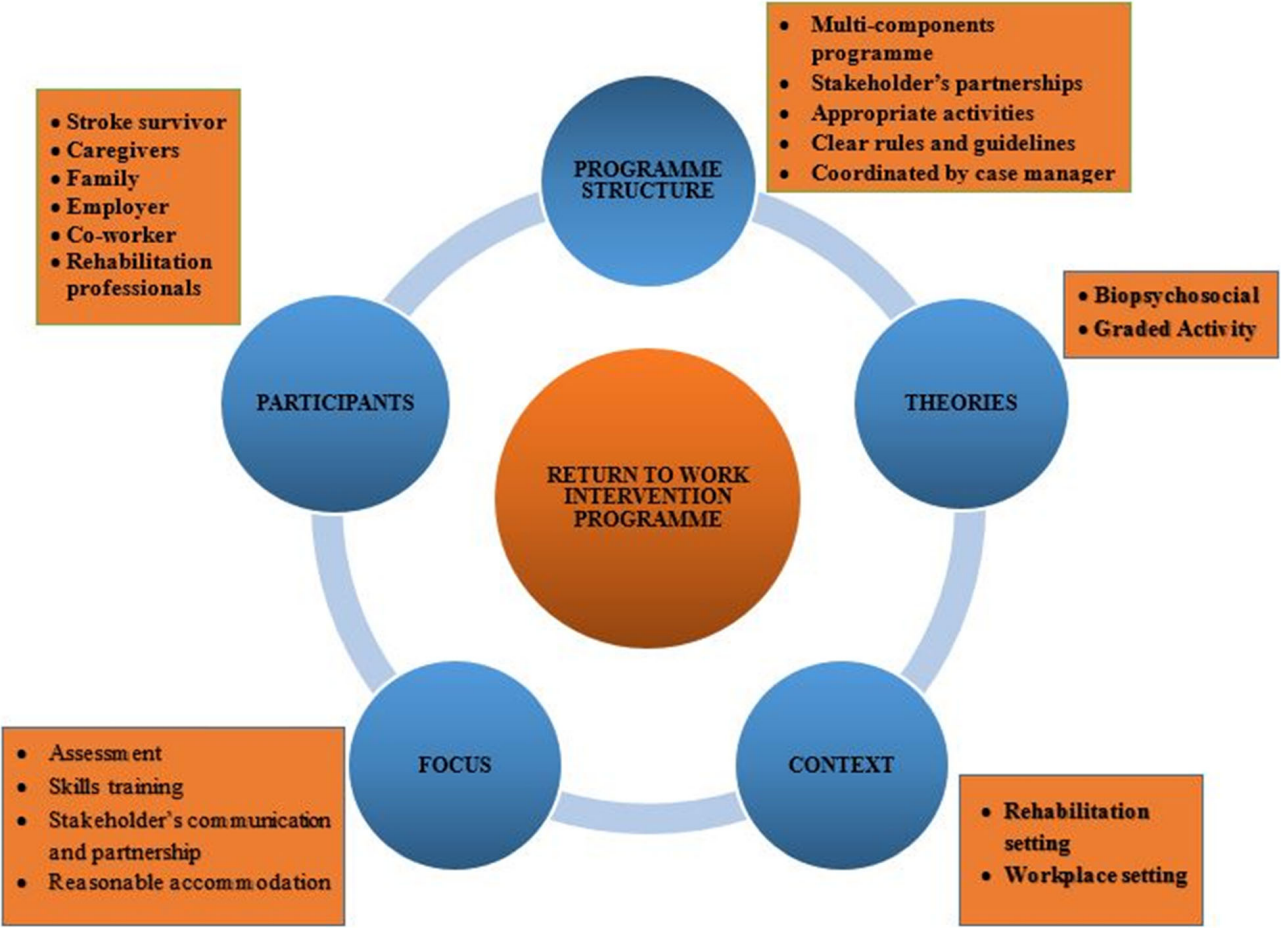
Concept map and design of Return to Work Intervention Programme

**Fig. 2:**
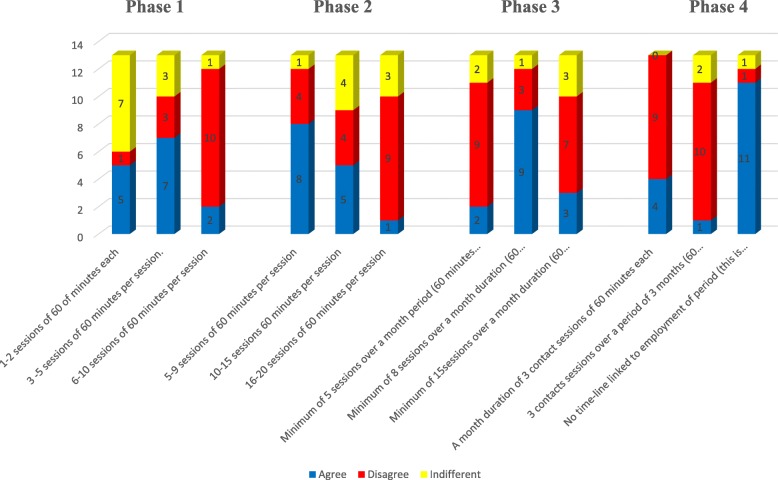
Response of panel of experts to the duration and frequency of treatment session in SReTWIP phases

The mean age and mean duration of practice of the experts was 44.73 ± 9.48 and 18.26 ± 8.71 years respectively. The demographic characteristics of the experts is summarized in Table 1. Three major themes emerged from the responses of the panel (Table 2). The concept map developed from the triangulation of findings from the previous two phases of the study is presented in Fig. 1. Five key areas contributed to the development of the SReTWIP into a deliverable and coherent RTW programme viz.: programme structure, participants, theories, context, and focus. The draft SReTWIP is presented in Table 3. The SReTWIP comprised four phases namely: an assessment phase (phase 1), a Work Intervention Training (WIT) phase (phase 2), Work Test Placement (WTP) phase, and the Clients Full Participation in Worker Role phase. The response rate to the second round Delphi was 86.7% as two experts who participated in the first round did not respond to the second round survey. The experts (*n* = 13, 100%) agreed that the structure and content of the SReTWIP should include work specific training and WTP phase while majority agreed that assessment (*n* = 10, 76.92%), non-work specific intervention (*n* = 11, 84.62%) and clients full work participation (*n* = 12, 92.31%) should be included. The experts however advised that the assessment phase should include goal setting and more of functional capacity evaluation that is conducted at the workplace compared to the use of work samples. Similarly, the experts suggested that stroke survivors’ self-awareness of strengths and weaknesses with regards to their ability to make accurate decisions about work performance, as well as a comprehensive review of the clients’ work integration should be considered at the final phase of the RTW programme. Consensus was reached on all the components of the assessment phase with the exception of its duration. Majority of the experts agreed on the inclusion of general functional skills training (*n* = 11, 84.62%); prevocational skills training (*n* = 9, 69.23%); and work hardening (*n* = 9, 69.23%) in the WIP phase of the SReTWIP. The experts consented to all intervention components included within the WTP phase and Clients Full Participation in Worker Role phase as well as their expected durations (Table 4). The experts’ responses to the duration and frequency of treatment sessions within the phases of the SReTWIP is presented in Fig. 2. Consensus was reached on four out of five suggested strategies for implementing the SReTWIP. There was also consensus on the time to commence the programme during stroke survivors’ recovery continuum. However, no consensus was reached by the experts on the period during stroke rehabilitation continuum when RTW should be commenced. In the third round, consensus was reached on all of the reviewed intervention components of the SReTWIP. Table 5 summarises the results of the third round of the Delphi survey.

**Table 1:** Demographic characteristics of panel of experts

ID	Age	Sex	Qualification	Country of Practice	Current Occupation	Years of Experience	Years of experience in SR/VR	Roles in SR and stroke VR
1	52	Male	M.Sc.	Nigeria	Clinical psychologist	27	17	A, I, M/F
2	55	Male	D.ClinPsy	UK	Neuropsychologist	31	20+	A, I, MF
3	39	Female	M.Sc.	Nigeria	Consultant OT (private practice)	14	6	A, I, R
4	33	Male	M.Sc.	Nigeria	OT clinician	7	7	A, I, CM, MF
5	31	Male	M.Sc.	UK	OT clinician	10	8	A, I, CM
6	37	Male	M.Sc.	Nigeria	OT clinician	13	10	A, I, MF
7	41	Male	M.Sc.	Canada	OT clinician	16	10	A, I
8	45	Female	Ph.D.	South Africa	Academia (PT)	23	8	A, I, MF
9	40	Male	M.Sc.	Australia	OT clinician	9	6	A
10	44	Male	Ph.D.	Nigeria	Academia (PT)	21	9	A
11	52	Male	M.Sc.	Nigeria	Academia (OT)	13	10	A, I, MF
12	43	Female	Ph.D.	Nigeria	Academia (PT)	20	13	A, I
13	45	Female	Ph.D.	Sweden	Academia and Private OT practice	20	10+	A, I, MF
14	45	Female	Ph.D.	Uganda	Academia (PT)	12	6	A, I, MF
15	69	Male	M.Sc	Nigeria	PT clinician	38	21	A, I, MF

*A* Assessment, *I* Intervention, *MF* Monitoring, and Follow-up, *CM* Case Management, *R* Researcher, *OT* Occupational therapist, *PT* Physiotherapist

**Table 2:** Themes from First Round of Delphi Survey

Theme	Categories	Quotes
**Theme 1:** **RTW intervention components addressing functional and work skills of stroke survivor**s	Assessment of functional capacity, work abilities and job analysis	*“Initial rehabilitation assessment, including assessment of the workers and employer’s needs- job and duties, expectations is the first thing needed” (Expert 9)*
	Basic functional skills training	“I*t depends on what the patient presents with. Impairments and activity limitations will be managed like any other stroke survivor focusing on cognitive function, activities of daily living (including instrumental) and mobility functional ability” (Expert 8)*
	Work skills training	*“This might need facilitating enrolment of the worker into a suitable programme to address gaps in skill requirements for the roles (Expert 9*). “*… include simulations of work environment within therapy sessions; Mobility - including endurance and speed; work tolerance and stamina, work conditioning” (Expert 7)*
	Adaptation of the work and work environment	“*… intervention should include adaptation of work setting- such as wheelchair ramps, support rails, elevators, wider corridors for wheelchair access” (Expert 5)* *“Phased return to work: gradually increase 1. Time at work, 2. Level of complexity of job functions.” (Expert 2)*
	Psycho-education of family, caregivers and employers	“Psycho-education for patient and employer will help” (Expert 2) “Liaison and advocacy with employer; family, caregiver and co-workers to ensure stability for survivor over the period of work re-entry” (Expert 12)
**Theme 2:** **Strategies for implementation**	Use of rehabilitation consultant or case manager	Use of rehabilitation consultant or case manager who might be an occupational therapist to facilitate the RTW plan. He will need to coordinate the RTW plan, complete assessments with key parties to identify their needs. (Expert 9)
	Use of team-based approach	*“Communication and collaboration among all stakeholders through team process, especially the rehabilitation practitioners working as a team during the return to work plan implementation. This might involve the employer if need be” (Expert 3)*
	Client centredness and individualized tailoring of intervention	*“The interventions should be client-centred, it should revolve around and be directly by the stroke survivor’s wish and be occupation focused.” (Expert 4)*
	Early implementation of RTW	*“Early implementation of the RTW plan immediately survivor is medically stable” (Expert 11)* *“Therapists should set long term goals that include possible work resumption for patients at onset of treatment. These goals* can be in achievable phases” (Expert 12)
**Theme 3:** **Essential elements of RTW intervention**	Objective assessment of work abilities and job analysis.	*Objective assessment of abilities is crucial. It is through objective assessment that RTW plan can meet the actual need of the client. (Expert 11)*
	Team work and communication among stakeholders	*Team based approach should be used in implementing interventions. (Expert 7)* *Clear communication in terms of treatment goals between therapist and workplace (Expert 11)*
	Work skills intervention	*Skills training especially work hardening, vocational counselling and career planning are important elements that should be in the program. (Expert 5)*

**Table 3:** Draft design of the Stroke Return to Work Intervention Programme

Phase	Description	Components	Duration
**Phase 1:** **Assessment Phase**	This is the first phase of the programme where preliminary interview is conducted with the clients and at the workplace to obtain information about the stroke survivor’s work ability and the job respectively	• Functional capacity evaluation • Job analysis; • Workplace ergonomic assessment	• 3–5 sessions of 60 min per session. OR • 6–10 sessions of 60 min per session
**Phase 2:** **Work Intervention Training phase**	This phase comprises of 2 focus areas where clinic based work interventions are provided to clients	**Focus area 1**: Non-work specific intervention/training section • General functional skills training	• 5–9 sessions of 60 min per session OR • 10–15 sessions 60 min per session OR • 16–20 sessions of 60 min per session
		**Focus area 2:** Work specific intervention/training section • Vocational counselling and career planning • Prevocational skills training (such as work competence, communication skills, work behavior, interviewing skills, education of legal aspects of work) • Formal education and training to improve job competitiveness • Work hardening that is inclusive of simulated task training	
**Phase 3:** **Work Test Placement**	This is a continuation of the previous phase. In this phase, other stakeholders in the RTW process of the stroke survivor are engaged. The interventions provided are both clinic and work based	Intervention in the phase include: • Education and preparation of clients, family, employer and co-worker about client’s abilities • Identification of suitable work opportunities/jobs • Work trials (practice of work skills in real work environment) • Job coaching and on-going support at the workplace• Ongoing environmental and ergonomic modification (structure, equipment, and organizational ergonomics).	• Minimum of 5 sessions over a month period (60 min each); OR • Minimum of 8 sessions over a month duration (60 min each) OR • Minimum of 15sessions over a month duration (60 min each)
**Phase 4:** **Clients Full Participation in the Worker Role**	This is the final phase of the RTW programme where the stroke survivor is fully reintegrated back to work	In this phase, • Clients is able (are trained) to make decisions about strengths and weaknesses • Clients can (are trained to) identify whether they require ongoing rehabilitation services for specific skills (if needed) • OT involvement is gradually decreased (tapered)	• A month duration of 3 contact sessions of 60 min each OR • 3 contact sessions over a period of 3 months (60 min each) OR • No time-line linked to employment of period (this is based on job availability) but OT contact is limited to 1–2 sessions per month over a period of 3 months
**Implementation Strategies and Period for the RTW intervention**	This section describes the strategies by which the different phases of the interventions are to be operationalized	**Implementation Strategies:** • Use of Multidisciplinary team-based approach • Use of Interdisciplinary team-based approach • Client-centered (client is involved in decision making process throughout the intervention). • Interventions are individually tailored to meet clients need • Use of case manager to coordinate return to work process	
		**Commencement of Rehabilitation Programme** • During out-patient rehabilitation • During in-patient rehabilitation • After out-patient rehabilitation • After the completion of medical intervention by physician • When client is independent in performing ADL tasks (self-care and mobility) • When client is independent in performing leisure activities • When client is fully reintegrated in the community (such as participation in social groups)

**Table 4:** Response of panel of experts to second round of Delphi survey

Phase	Agree n (%)	Disagree n (%)	Indifferent n (%)
**Phase 1 components: Assessment**			
• Functional capacity evaluation	13	0	0
• Job analysis	13	0	0
• Workplace ergonomic assessment	13	0	0
**Phase 2 components: Work Intervention Training**			
*Focus area 1: Non-work specific intervention/ training session*			
• In this focus area, general functional skills training are provided for the stroke survivor	11 (84.6)	2 (15.4)	0 (0.0)
*Focus area 2: Work specific intervention/ training session*			
• Prevocational skills training (such as work competence, communication skills, work behavior, interviewing skills, education of legal aspects of work, commuting to and from work)	9 (69.2)	2 (15.4)	2 (15.4)
• Vocational counselling and career planning	8 (61.5)	1 (7.7)	4 (30.8)
• Formal education and training to improve job competitiveness	6 (46.1)	3 (23.1)	4 (30.8)
• Work hardening that is inclusive of simulated task training	9 (69.2)	2 (15.4)	2 (15.4)
**Phase 3 components: Work Test Placement**			
• Education and preparation of clients, family, employer and co-worker about client’s abilities	9 (69.2)	0 (0.00)	4 (30.8)
• Identification of suitable work opportunities/jobs	9 (69.2)	2 (15.4)	2 (15.4)
• Work trials (practice of work skills in real work environment)	11 (84.6)	2 (15.4)	0 (0.0)
• Job coaching and on-going support at the workplace	10 (76.9)	1 (7.7)	2 (15.4)
**Phase 4 components: Clients Full Participation in the Worker Role**			
• Clients is able (are trained) to make decisions about strengths and weaknesses	12 (92.3)	1 (7.7)	0 (0.0)
• Clients can (are trained to) identify whether they require ongoing rehabilitation services for specific skills (if needed)	12 (92.3)	1 (7.7)	0 (0.0)
• Rehabilitation professional involvement is gradually decreased (tapered)	12 (92.3)	1 (7.7)	0 (0.0)
• Clients is able (are trained) to make decisions about strengths and weaknesses	12 (92.3)	1 (7.7)	0 (0.0)
**Implementation Strategies of RTW intervention**			
• Use of Multidisciplinary team-based approach	4 (30.8)	8 (61.5)	1 (7.7)
• Use of interdisciplinary team-based approach	11 (84.6)	1 (7.7)	1 (7.7)
• Client-centered (client is involved in decision making process throughout the intervention)	11 (84.6)	2 (15.4)	0 (0.0)
• Interventions are individually tailored to meet clients need	11 (84.6)	2 (15.4)	0 (0.0)
• Use of case manager to coordinate return to work process	9 (69.2)	3 (23.1)	1 (7.7)
**When to commence the programme during stroke rehabilitation**			
• During out-patient rehabilitation	7 (53.8)	4 (30.8)	2 (15.4)
• During in-patient rehabilitation	7 (53.8)	5 (38.5)	1 (7.7)
• After out-patient rehabilitation	8 (61.5)	3 (23.1)	2 (15.4)
• After the completion of medical intervention by physician	8 (61.5)	2 (15.4)	3 (23.1)
**When to commence the programme during recovery continuum**			
• When client is independent in performing ADL tasks (self-care and mobility)	12 (92.3)	1 (7.7)	0 (0.0)
• When client is independent in performing leisure activities	3 (23.1)	9 (69.2)	1 (7.7)
• When client is fully reintegrated in the community (such as participation in social groups)	2 (15.4)	8 (61.5)	3 (23.1)

**Table 5:** Response of panel of experts to third round of Delphi survey

Items	n (%)	Comments
**Duration of RTW Assessment phase**		
How many sessions?		
• Minimum of 1–2 sessions	3 (23.1)	
• Minimum of 3–5 sessions	10 (76.9)	
How long should each Assessment Session take?		
• 30 min	1 (7.7)	
• 45–60	12 (92.3)	
**Duration of WIT phase**		
How many sessions?		As RTW intervention is client-centred, the frequency and duration of intervention will depend on personal and clinical factors of the client.
• Minimum of 5–9 sessions	11 (84.6)	
• Minimum of 10–15 sessions	2 (15.4)	
How long should each Assessment session take?		
• 30 min	1 (7.7)	
• 45–60 min	12 (92.3)	
**Relevance of intervention components within WTP**		
Vocational counselling and career planning		
• Relevant	13 (100.0)	
• Not relevant	0 (0.0)	
Formal education and training to improve job competitiveness		
• Relevant	9 (69.2)	
• Not Relevant	4 (30.8)	
**Implementation Period of the RTW intervention**		
During out-patient rehabilitation		Essential to go side by side with other treatment schedules at the onset
• Yes	12 (92.3)	
• No	1 (7.7)	
After out-patient rehabilitation		
• Yes	3 (23.1)	
• No	10 (76.9)	
After the completion of medical intervention by physician		
• Yes	4 (30.8)	
• No	9 (69.2)	

**Fig. 3:**
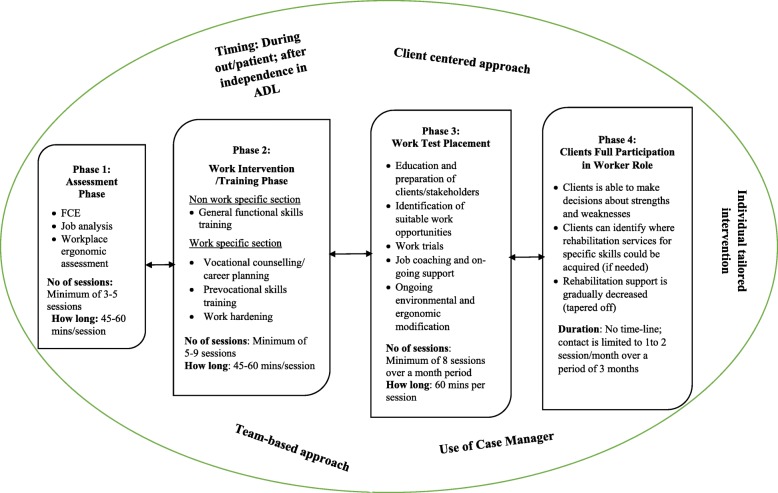
Stroke Return to Work Intervention Programme (SReTWIP)

The developed SReTWIP is presented in Fig. 3. The SReTWIP comprised four interconnected phases of interventions that will span 12 weeks. The programme commences with an assessment phase (phase 1) where comprehensive work ability assessment, workplace ergonomic assessment and goal settings are conducted for the stroke survivor. The phase is to be initiated during out-patient rehabilitation, after the client is independent in performing ADL. The phase is to be conducted over a minimum of three to five sessions after which the WIT phase will commence. In the WIT phase, work and non-work specific intervention are provided for the stroke survivors in the clinic. These include: general functional skills training, vocational counselling and career planning as well as prevocational skills training. The WIT phase is to be conducted over a minimum of 5 to 9 treatment sessions. When competency has been achieved in the second phase by the stroke survivor, the client proceeds to a third phase, which is the WTP phase. In this phase, other stakeholders in the RTW process are engaged. The interventions provided are both clinic and work based, to be conducted over a minimum of 8 treatment sessions of 60 min each. The final phase of the SReTWIP, Clients Full Participation in Worker Role, envisions that the stroke survivor would have achieved competencies in varying work aspects of the previous three phases. During this stage, the stroke survivor would be encouraged to undergo self-reflection on their ability to participate in their occupational role as a worker. The client then synthesizes and internalizes the actions undertaken and skills acquired during the previous phases. In addition, stroke survivors are encouraged to make decisions on strengths and weaknesses as well as choice of rehabilitation services best suited to the specific skill that they require. The involvement of core rehabilitation specialist is gradually tapered during this phase. The entire process of the SReTWIP will be individually tailored to meet the needs of the stroke survivor and implemented by an IDT that will include the occupational therapists and physiotherapist as key members. Equally, the stroke survivor is expected to be involved in the decision making process throughout the duration of the SReTWIP. And finally, the programme is to be coordinated by a case manager who will be a member of the interdisciplinary team.

## Discussion

The SReTWIP is the first VR programme that identified the essential RTW components and provided a clear pathway for implementing RTW intervention for stroke survivors. On completion of the Delphi study, minor changes were effected in the first three phases of the programme and in the programme implementation strategies. Firstly, the “duration” of the various phases were renamed to “frequency and duration” in order to accommodate for the timing (how long) and the numbers of sessions (how often) required for each phase of the programme. This provided clear information regarding the frequency of the interventions and served as a step for the proper quantification of the various intervention phases to ensure future fidelity assessment [17]. The panel of experts established the relevance of functional capacity evaluation, job analysis and workplace ergonomic assessment of the stroke survivor during VR. Comprehensive work assessment helps to determine the stroke survivors’ eligibility for services as well as the nature and scope of interventions to be included during the subsequent phases of the SReTWIP [18]. Holistic understanding of the stroke survivor’s work capacities and barriers is important for delivering the SReTWIP effectively. The inclusion of goal setting as an integral part of the assessment phase of the SReTWIP could enhance communication and collaboration within rehabilitation teams [19–21] as well as improve patient motivation and engagement in the other phases of the SReTWIP programme [22]. Although in most literature, goal setting during stroke rehabilitation has been generally referred to as a single activity that is isolated from other rehabilitation processes [19, 23, 24]. Plant and Tyson [25], on the contrary asserted that goal setting process as well as patient’s assessment during stroke rehabilitation are clearly interlinked. The above authors suggested the integration of the two processes in long term post stroke rehabilitation.

Furthermore, the inclusion of non-work specific and work specific sections in the WIT phase were indicated by the experts. Intervention contents within this phase comprised vocational counselling and career planning, prevocational skills trainings work hardening and work simulation task training as well as general functional skills training which may be delivered as a single or multicomponent form. As work disability after stroke could result from a combination of difficulties experienced at stroke survivor’s personal and societal levels based on the biopsychosocial framework of the ICF [26], work skills training in the WIT phase will address skills deficit from the stroke event. It will further help to build self-confidence and competence of the stroke survivor before re-entering the workplace [27]. On the other hand, formal education and training to improve job competitiveness was excluded as a component of the WIP phase. Although it was highlighted by the experts that upgrade of skill sets required for work performance may be needed, it was however opined that these would not be conducted by rehabilitation teams involved in the RTW process of the stroke survivor. Thus, formal education and upgrading of skill sets were excluded from the SReTWIP by the experts.

The WTP phase of the SReTWIP recognized the need for a fit between the work abilities of the worker and the job through work trials, identification of suitable work opportunities, ergonomic and environmental modification in order to encourage work participation [28]. Reasonable accommodation in the form of ergonomic and environmental modification provides opportunity for RTW efforts, especially in situations where restoration of deficits in work skills may otherwise not be possible. Such ergonomic and environmental modification has been adduced to facilitate not only work resumption but also job retention among stroke survivors [29]. Similarly, job coaching, a one on one intensive support and feedback to achieve competence on the job by stroke survivors within the WTP phase could effectively enhance work retention [3].

With regards to the implementation strategies, the multi-disciplinary team-based approach (MDT) was excluded, as experts felt that the interdisciplinary approach (IDT) was more suited for delivering the RTW intervention. Although MDT has been associated with improvements in the quality of stroke care by policy makers and clinical guideline developers in the literature [30–33], considering the context in which the RTW would take place, the experts felt that the conceivable value of integrated team action may not be achieved using MDT. Rather the IDT was considered as a more practicable approach as it allows team members to perform activities toward a common goal such as RTW, and accept the additional obligation of group effort for clients. Disciplinary articulation provided within the IDT could have further informed the position taken by the experts since the IDT enables team members to have an understanding of each other’s roles and identifies where overlap occurs [33, 34]. As IDT allows RTW team members to work as equals, with reverence for the expertise and knowledge provided by each team member, it will therefore facilitate a more cohesive and efficient approach to collaborative working among the RTW team when implementing the SReTWIP. Furthermore, IDT in stroke management has been attributed to ease prompt information exchange and facilitates early interventions, as well as an effective approach in longer term rehabilitation in community settings [35, 36]. The use of case managers to oversee the SReTWIP was considered an important strategy in achieving the goals of the programme by the experts. VR coordinators and case managers have been acknowledged as vital players in the success of RTW process [3, 37]. Utilization of case managers when implementing the SReTWIP will ensure that the workplace is set as the core of RTW plan. With this, rehabilitation activities can be implemented and progressively centralized within the stroke survivors’ workplace with a focus on the job to be returned to by the survivor [37].

## Conclusion

The SReTWIP is the first step in developing a VR pathway that could ultimately enhance the RTW rates and quick resumption of the worker role of stroke survivors. The stroke survivor can move along the different phases of the SReTWIP after achieving competency in a preceding phase. Future work will include a feasibility study with other key stakeholders involved in RTW such as employers, informal caregivers and the stroke survivors before its implementation.

## Data Availability

The datasets used and/or analyzed during the current study are available from the corresponding author on reasonable request.

## Abbreviations

ICF: International Classification of Functioning, Disability and Health
IDT: Interdisciplinary Team
MDT: Multi-disciplinary Team
MSD: Musculoskeletal dysfunction
OT: Occupational therapist
PT: Physiotherapist
RTW: Return to Work
SReTWIP: Stroke Return to Work Intervention Programme
TBI: Traumatic Brain Injury
VR: Vocational rehabilitation
WIT: Work Intervention and Training
WTP: Work Test Placement
